# Organoid scaffold materials: research and application

**DOI:** 10.3389/fbioe.2025.1637456

**Published:** 2025-07-18

**Authors:** Ziwei Zhang, Maoshu Zhu, Haiqing Luo, Fanwei Zeng, Zhongquan Qi

**Affiliations:** ^1^ Medical College, Guangxi University, Nanning, Guangxi, China; ^2^ Central Laboratory, The Fifth Hospital of Xiamen, Xiamen, Fujian, China; ^3^ Xiamen Mogengel Biotechnology Co., Ltd., Xiamen, Fujian, China; ^4^ State Key Laboratory of Physical Chemistry of Solid Surfaces, Collaborative Innovation Center of Chemistry for Energy Materials, College of Chemistry and Chemical Engineering, Xiamen University, Xiamen, Fujian, China; ^5^ Fujian Provincial Sperm Bank, Fujian Maternity and Child Health Hospital, Fuzhou, Fujian, China

**Keywords:** organoid scaffolds, principles, applications, culture, categorize

## Abstract

In recent years, organoid research has witnessed remarkable progress, yet significant challenges remain in organoid construction. As fundamental architectural frameworks, organoid scaffolds play a pivotal role in facilitating three-dimensional tissue morphogenesis by delivering crucial biochemical and mechanical signals during *in vitro* organoid development. A systematic examination of scaffold functions in organoid culture systems, coupled with a critical assessment of different scaffold modalities, not only deepens our understanding of organoid biology and their microenvironment but also provides valuable insights for next-generation scaffold design. This review elucidates the fabrication principles and applications of organoid scaffolds, delineates their functional significance in organoid culture, categorizes existing scaffold systems with comparative analysis of their respective merits and limitations, and concludes with perspectives on future research directions in scaffold development.

## 1 Introduction

Organoids, three-dimensional cell cultures derived from embryonic or adult stem cells *in vitro*, exhibit histological characteristics similar to human organs and can partially replicate their physiological functions. Organoids induced from human pluripotent stem cells (hPSCs) reproduce key features of human embryonic development, providing an unprecedented view of early human development. Organoids derived from adult stem cells, including animal organoids, human normal tissue organoids, and tumor organoids, have shown significant value in disease mechanism research, new drug development, and regenerative medicine. The external environment required for the growth of organoids mainly includes culture medium and scaffold materials. The culture medium provides nutrition for organoids and regulates the directional differentiation of organoids. Organoid scaffolds mimic the mechanical and biochemical properties of tissues, providing a suitable microenvironment for organoid growth and ensuring the normal progression of their life activities. However, there are still many challenges in the success rate, tissue simulation, functional stability, and structural order of organoid modeling. To solve the dilemma in the current research, researchers have adjusted and optimized the external environment of organoid growth in multiple directions. This article mainly introduces the exploration of organoid scaffolds, from the complex composition of Matrigel scaffold and decellularized extracellular matrix (dECM) hydrogel scaffold to the specific composition of recombinant protein and peptide hydrogel scaffold and synthetic hydrogel scaffold. Researchers hope to study the role of each scaffold component in organoid culture. Adjusting the mechanical and biochemical properties of scaffolds to prepare scaffolds more suitable for organoids will provide strong support for the application of organoids in disease research, drug research and development, precision medicine, and regenerative medicine. This review aims to elaborate on the principle of scaffold preparation, summarize the important role of scaffolds in organoid culture, summarize the various scaffolds currently used for organoid culture, and compare their advantages and disadvantages. Finally, relevant insights on the future research direction of organoid scaffolds were proposed.

## 2 Introduction to organoid scaffolds

### 2.1 Classification of organoid scaffolds

Stimuli-triggered hydrogel matrices for organoid engineering exhibit tri-modal responsiveness, demonstrating programmable structural transitions under thermal, pH, or optical excitation. Among them, temperature-sensitive hydrogel scaffolds are widely used in organoid culture. Temperature-sensitive hydrogels possess a unique molecular architecture containing both hydrophilic and hydrophobic functional groups. These dual-character components undergo dynamic intramolecular and intermolecular interactions with water molecules in response to thermal variations, ultimately triggering structural reorganization of the hydrogel’s three-dimensional network and consequent volumetric phase transitions (Wang et al., 2019). This thermal responsiveness is primarily attributed to a characteristic thermotropic phenomenon observed in certain polymer solutions, where aqueous solubility decreases progressively with temperature elevation. Upon reaching a critical threshold temperature, the system undergoes phase separation manifested by solution turbidity, while cooling below this transition point restores homogeneous transparency - a thermodynamic behavior formally designated as the Lower Critical Solution Temperature (Iyer et al., 2013). On the contrary, a part of the polymer is dissolved above a certain temperature. Below this temperature, the polymer solution undergoes phase separation and precipitation, called the upper critical dissolution temperature (Seuring and Agarwal, 2012). The low critical dissolution temperature and the upper critical dissolution temperature are important parameters for the interconversion of solutions in transparent and opaque states. Thermosensitive hydrogel scaffolds for organoid culture are usually regulated by a low critical dissolution temperature. For example: Matrigel, Mogengel, and BME, all derived from EHS cells, exist in solution form at 4°C and convert to gel from 22°C to 35°C. Like these hydrogels, the thermosensitive dECM hydrogel scaffolds were in solution from 4°C to 8°C and polymerized into the gel from 37°C (Zhu et al., 2023). In contrast, polyisocyanate (PIC)-based composite hydrogel scaffolds can form gels at 18°C (Kouwer et al., 2013).

Unlike temperature-sensitive hydrogels, pH-sensitive hydrogels generally contain weakly acidic or basic groups. After the environmental pH value or ionic strength changes, due to the difference in internal and external concentration, the gel water absorption swells or contracts, and the hydrogen bond formed between the polymers will ionize, causing discontinuous swelling changes in volume (Hendi et al., 2020). The pH-sensitive hydrogel scaffolds used for organoid culture include polyethylene glycol (PEG)-based hydrogels (Chrisnandy et al., 2022), hyaluronic acid (HA) hydrogels (Chen Y. et al., 2023), self-assembling peptide hydrogels (SAPHs) (Treacy et al., 2023), etc. The preparation methods of pH-responsive scaffolds include: 1. Crosslinking polymerization, including chemical initiator-initiated monomer crosslinking polymerization and crosslinking by radiation technology; 2. Graft copolymerization involves combining two chain segments with special properties that are typically incompatible, such as hydrophilic and hydrophobic, or acidic and basic. The characteristics of the resulting gel depend on the composition and length of both the backbone and side chains, as well as the number of side chains present; 3. Interpenetrating polymer technology; 4. Cross-linking of water-soluble polymers (Zhang and Wu, 2023).

Due to the advantages of dose adjustability, wavelength orthogonality, and spatiotemporal controllability, light has become a highly potential stimulation means to regulate the properties of hydrogels (Homma et al., 2021). Photosensitive hydrogels consist of a polymeric network and a photoreactive group, usually containing photochromic chromogenic groups that can undergo physical or chemical changes in response to light signals. There are three main mechanisms for the reaction of photosensitive hydrogels: 1. The photosensitive molecules in the photosensitive material convert light energy into heat energy, thereby increasing the temperature of the material. When the internal temperature of the gel reaches the phase transition condition, the gel will respond (Xing et al., 2016). 2. Photoresponsive hydrogels undergo programmable volumetric transitions through light-triggered ionic modulation. Upon photoactivation, photolabile moieties within the polymer network undergo photolytic cleavage, liberating ionic species that disrupt the Donnan equilibrium. This osmotic gradient drives compensatory mass transport phenomena, where controlled ion flux across the hydrogel-solvent interface induces reversible swelling/deswelling behavior via electrostatic repulsion force modulation (Dehghany et al., 2018). 3. Photochromic molecules are introduced into the hydrogel material of the polymer backbone as side groups or cross-linking agents. Due to the photosensitivity of these chromophores, their physicochemical properties, such as dipole moments and geometry, as well as the shape, structure, and properties of the macroscopic hydrogels, change due to expansion and contraction (Ji et al., 2020). Photosensitive hydrogels are also commonly used for organoid cultures, such as allyl sulfide hydrogel for intestinal organoids (Hushka et al., 2020); Broguiere et al. used a two-photon patterning technique to guide axons in a hyaluronic acid matrix by photopatterning nerve growth factors (Broguiere et al., 2020).

Organoid scaffolds can precisely regulate their mechanical and biochemical properties through different response mechanisms (temperature, pH, and light), which cause changes in the structure or properties of the materials. Thermosensitive hydrogels can precisely control the mechanical and biochemical properties of scaffolds through the use of temperature-responsive polymers. In terms of mechanical properties, when the temperature changes, the polymer chains undergo reversible hydrophilic-hydrophobic phase transitions, thereby dynamically adjusting the mechanical properties of the scaffold such as viscoelasticity and porosity (Zhao J. et al., 2022). In terms of biochemical properties, thermosensitive hydrogels can act as intelligent delivery carriers, and through temperature-responsive swelling and contraction behaviors, achieve controlled release of loaded growth factors, drugs, and other bioactive substances. This dynamic release characteristic not only avoids the burst release effect but also provides a continuous and stable biochemical microenvironment for organoids (Hasani-Sadrabadi et al., 2020). Unlike thermosensitive hydrogels, pH-sensitive hydrogels achieve dynamic regulation of the mechanical and biochemical properties of the scaffolds through polymer networks containing ion groups. In terms of mechanical properties, changes in environmental pH can dynamically alter the ionization state of the polymer chains, and by adjusting the electrostatic repulsion between molecular chains and the swelling equilibrium, precisely control the mechanical properties of the scaffold (Qiu and Park, 2001). In terms of biochemical property regulation, pH-dependent surface charge changes can not only respond to environmental stimuli to achieve controlled release of bioactive substances but also promote cell adhesion and proliferation by altering the surface electrical properties of the material (Ahmadian et al., 2021; Gupta et al., 2002). Different from the aforementioned two types of hydrogels, photosensitive hydrogels achieve regulation of the mechanical and biochemical properties of the scaffolds by introducing photosensitive groups. In terms of mechanical property regulation, ultraviolet or visible light irradiation can trigger photo-crosslinking or photolysis reactions, dynamically adjusting the crosslinking density of the polymer network, thereby achieving reversible regulation of the mechanical properties of the hydrogel such as viscoelasticity and network structure (Tsegay et al., 2022). In terms of biochemical property regulation, the photosensitive groups in the hydrogel can achieve light-controlled release of bioactive substances. That is, under specific wavelength light irradiation, the photosensitive groups in the hydrogel react, causing changes in the hydrogel structure and releasing the bioactive substances encapsulated within, thereby regulating the life activities of organoids (Zhao X. et al., 2022). Through the above-mentioned external stimulus-response mechanisms, organoid scaffolds can dynamically regulate their mechanical and biochemical properties. Therefore, on the basis of clearly classifying the response modes of the scaffolds, further exploration of the mechanical and biochemical properties of organoid scaffolds is of great significance for an in-depth understanding of the scaffold-cell interaction mechanism and optimization of the organoid culture system.

### 2.2 Characteristics of scaffold materials

Organoid scaffolds have both mechanical and biochemical properties. Mechanical properties provide structural support for organoids, while biochemical properties provide bioactive substances required for the life activities of organoids.

#### 2.2.1 Mechanical characteristics

Under the stimulation of the external environment (temperature, pH, light, etc.), the organoid scaffold undergoes physical or chemical cross-linking to form a stable three-dimensional network structure. There are many components involved in the formation of 3D network structures, such as collagen, fibrin, laminin, and hyaluronic acid (HA). The three-dimensional complex network of organoid scaffolds can provide structural support for organoids. At the same time, the pores of the network can act as transport channels for nutrients to support the life activities of organoids. In addition, the pore size of the scaffold affects cell growth, migration, and proliferation (Paul et al., 2017; Coluccio et al., 2020). For example, a recent study investigated the effect of scaffold pore size on organoid growth and showed differences in endometrial organoids cultured on three organoid scaffolds with different pore sizes, with optimal growth conditions of 101 ± 38 microns (Abbas et al., 2020).

In the 3D environment, organoids are subject to physical limitations in addition to scaffold network structure, including scaffold plasticity, viscoelasticity, and degradability (Saraswathibhatla et al., 2023). These properties show a coupled relationship in the process of cell-matrix remodeling: in a viscoelastic and plastic matrix, cell activity can affect pore size (Wisdom et al., 2018), matrix degradation can directly affect its viscoelasticity (Schultz et al., 2015), and changes in matrix structure may affect its viscoelasticity and degradability simultaneously. In the process of organoid culture, these characteristics have important effects on the development, growth, and proliferation of organoids.

The viscoelasticity of scaffolds plays an important role in cell adhesion, proliferation, differentiation, and migration (Huang et al., 2019). For example, recently, researchers have developed a viscoelastic scaffold material that can be used as a scaffold to grow Madin-Darby canine kidney (MDCK) cells and successfully generate cystic organoids. It was found during culture that the polarity of the cyst could be modulated by stress relaxation of the material. For example, when replacing 14 nt SRC with 18 nt SRC (a stress-relaxing crosslinker), apical polarity can be increased to 90% (±6%) (Peng et al., 2023).

The plasticity of organoid scaffolds has an important impact on the transport of organoid nutrients (Rothenbucher et al., 2021), the growth and differentiation of organoids (Shan et al., 2024), and the structural stability of scaffolds. For example: Recently, studies have evaluated the effect of matrix plasticity on breast cancer cell invasion and migration. They developed a hydrogel scaffold with adjustable plasticity, fabricated by mixing a recombinant basement membrane with alginate. The plasticity of the scaffold was changed by applying a 100 Pa creep stress for 1 h. The results showed that cells in the high-plasticity (30%) scaffold could migrate in a protein-independent manner, while cells in the low-plasticity (10%) scaffold mostly did not migrate (Schultz et al., 2015).

The degradability of scaffolds has an important impact on organoid growth, function, and cell-to-cell interactions. For example, a recent study showed differences in the size of the trachea in lung organoids cultured using organoid scaffolds with different degradation rates, with the largest organoids cultured with scaffolds with moderate degradation rates. In addition, the degradation process of polymers in scaffolds can effectively remove the polymers as substrates for organoid development, which further affects the proliferation and maturation of organoids (Kivijarvi et al., 2022).

#### 2.2.2 Biochemical characteristics

Bioactive substances contained in the organoid scaffold include proteins (collagen, fibronectin, laminin, elastin), peptides (RGD), glycosaminoglycans (GAGs) (HA, chondroitin sulfate) and cytokines (epidermal growth factor (EGF), insulin-like growth factor 1 (IGF-1), fibroblasts) required for organoid growth Growth factors (FGF)), which together regulate various behaviors of organoids, such as cell adhesion, proliferation, differentiation and migration. The functional deficiency of bioactive components in hydrogel matrices critically compromises cellular adhesion capacity, ultimately triggering apoptotic cell death due to insufficient substrate-cell interactions (Grafahrend et al., 2008).

Collagen, laminin, RGD, etc. can bind to cells through integrins or related receptors to ensure adhesion and information transfer between cells and scaffolds (Wisdom et al., 2018). For example, recently, researchers successfully fabricated a fully synthetic PEG hydrogel scaffold by adding proteins and peptides related to the growth of pancreatic organoids to the scaffold. They showed that the proteins and peptides (laminin, collagen, fibronectin, PHSRN-K-RGD, GFOGER, BM) incorporated into the scaffold were associated with the adhesion and growth of pancreatic cancer organoids, and the scaffold supported the interaction between pancreatic cancer organoids and the (extracellular matrix) ECM (Below et al., 2022).

GAGs can interact with a variety of molecules, including proteases, growth factors, cytokines, and adhesion molecules, and thus, can mediate many physiological processes, such as protein function, cell adhesion, and signaling (Shi et al., 2021). For example, in the drug sensitivity test of pancreatic ductal adenocarcinoma organoids, it was found that the interaction of HA with the CD44 receptor would increase the expression of drug efflux transporter, which would cause drug resistance (LeSavage et al., 2024).

EGF, IGF, FGF, and other signaling molecules can regulate cell growth, proliferation, and migration (Kluiver et al., 2024; Belair et al., 2018). It has been shown that the combination of IGF-1 and FGF-2 can promote the growth of most p38i-sensitive organoids. For example, patient-derived ulcerative colitis organoid lines grew in cultures containing both growth factors and barely grew in other culture conditions (Fujii et al., 2018).

#### 2.2.3 Mechanical and biochemical properties interact

Organoid scaffold is a complex three-dimensional network, and its mechanical and biochemical properties interact and influence each other to regulate the behavior of cells (Hoffmann et al., 2019). For example, researchers cultured kidney organoids on alginate gel scaffolds to explore the effect of the scaffold microenvironment on kidney organoids. They regulated scaffold stiffness and viscoelasticity by varying the concentration of Ca^2+^ crosslinker and the molecular weight of alginate and tested the effects of Ca^2+^ and RGD on renal organoids. The results showed that the viscoelasticity of the scaffold affected the spatial distribution and morphology of the kidney organoids (the organoids in the fast-relaxation hydrogel were more widely distributed); RGD-mediated mechanical interactions can affect the viscoelasticity of the scaffold, and thus the nephron density in the organoid; The release of Ca^2+^ from the hydrogel decreases the ratio of glomerular to tubular nephron segments; The degree of deformation of the hydrogel-organoid interface can modulate the length and morphology of nephron segments in renal organoids (Nerger et al., 2024).

#### 2.2.4 Cell-scaffold interaction

Cells adhered to the scaffold and generated internal stress, which changed the structure and arrangement of the scaffold and guided cell migration. Scaffold mechanical signals can be converted into biochemical signals through mechanical transduction to further regulate cell behavior (Anguiano et al., 2020). For example, recently, researchers incorporated electrospun dextran vinyl sulfone fibers and basement membrane binder (BMB) into PEG hydrogel to produce an oocyte-based scaffold. The scaffold not only has the function of ECM isolation peptide BMB, but also provides support for the deposition and remodeling of ECM, and has a fiber structure similar to natural ECM. The results showed that the growth of follicles and oocytes cultured on the scaffold was significantly improved, and the ECM deposition of laminin, perlecan, and type I collagen was increased. In addition, it has been noted that ECM deposited on fibers has a positive effect on follicle and oocyte growth. These ECM components can promote follicular gonadal hormone secretion, which further promotes oocyte growth and drives cell aggregation (Nason-Tomaszewski et al., 2024).

**FIGURE 1:**
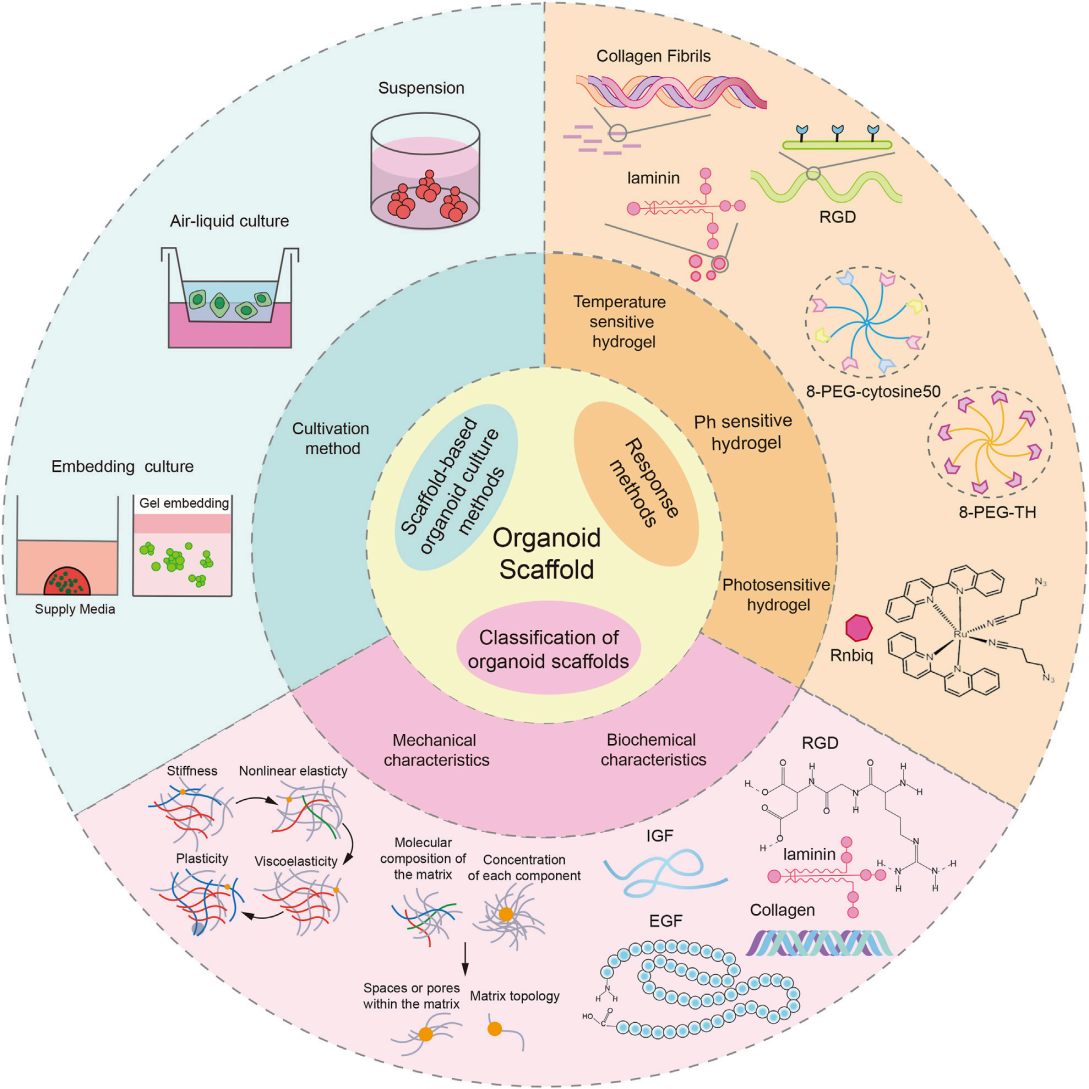
Introduction to organoid scaffolds. The response mode and characteristics of the organoid scaffold and the organoid culture mode based on the organoid scaffold were demonstrated. This picture was created using the drawing software BioRender.

In summary, the mechanical and biochemical properties of organoid scaffolds interact to provide support and necessary bioactive substances for organoids. At the same time, the microenvironment formed by organoids in the scaffold will also react to the scaffold, making it more suitable for the growth and functional maturation of organoids. This dynamic interaction is important for organoid formation and growth (Figure 1).

### 2.3 Scaffold-based organoid culture methods

The mechanical properties, biochemical characteristics, and response patterns of organoid scaffolds are key factors in regulating the formation, development, and maturation of organoids. Therefore, studying scaffold-based organoid culture systems and deeply analyzing the mechanical features, biochemical composition and dynamic responses of scaffolds to different culture systems are crucial for optimizing organoid culture models.

The methods for culturing organoids using hydrogels as scaffolds primarily encompass gel embedding, air-liquid interface culture, and suspension culture. Among these techniques, gel embedding is the most prevalent, comprising two principal approaches: the dome method and the cover culture method. Recently, some researchers have conducted a standardized study on the dome culture of organoids. They used Matrigel as an organoid scaffold and found that the temperature was 4°C for the best uniformity of the gel droplets, and the inoculum was 25 μL for the best organoid growth (Moon Hyun et al., 2024). The cover culture method is also frequently used in organoid cultures. Gel was added to the bottom of the culture plate, and after the gel had been set, cells were spread on the gel and covered with a layer of gel above the cells to form a three-layer culture system. This method is not only suitable for tumor organoid culture but also can be used to co-culture tumor organoids and immune cells to evaluate the killing ability of different types of immune cells on tumor and infected organoids (Ota et al., 2024; Sharma et al., 2021). For organoid culture at the air-liquid interface, firstly, the diluted gel was added to a Transwell chamber, and after it solidified, gel suspension containing organoids was added. After the suspension had solidified, the Transwell chamber was placed in a Petri dish and a medium suitable for organoid growth was added to it, thus forming an air-liquid culture system (Neal et al., 2018). In addition to the above two culture methods, organoids can also be cultivated by suspension culture. In this method, organoids are suspended in a medium containing 3%–10% matrix glue or other 5% matrix preparations, and the organoids are stirred by pipetting to prevent them from sticking to the walls of the culture vessel. For example, Yumiko et al. successfully cultured colorectal organoids and CRC (colorectal cancer) organoids using suspension culture. The experimental results showed that the suspension culture organoids were similar in morphology to the organoids cultured by the traditional embedding method. Furthermore, the generated CRC organoids exhibited histological features and genetic heterogeneity similar to primary CRC (Hirokawa et al., 2021).

It is worth noting that different culture methods have distinct requirements for scaffold properties. In long-term gel-encapsulated culture, the degradation rate of the scaffold needs to be well-matched with the tissue regeneration process too rapid degradation can lead to structural collapse, while too slow degradation can impede the remodeling of the extracellular matrix (Place et al., 2009; Lutolf and Hubbell, 2005). For air-liquid interface culture, the scaffold should possess biochemical characteristics that promote epithelial polarity growth (such as collagen and laminin modification) and a porous structure that facilitates the exchange of gases and nutrients (Chen and Schoen, 2019). In suspension culture systems, the viscosity of the scaffold must be precisely controlled; too low viscosity may cause cell sedimentation and aggregation, while too high viscosity can restrict the diffusion of oxygen and nutrients (Hirokawa et al., 2021). Therefore, for different culture methods, the mechanical and biochemical properties of the scaffold need to be precisely designed and optimized according to their specific requirements.

The organoid scaffolds enhance the regulatory ability of the cultivation system for complex microenvironments through their dynamic responses to external stimuli. Among them, the thermosensitive hydrogel scaffolds demonstrate unique advantages in the embedding method: when the minimum critical dissolution temperature is set between 30°C and 32°C, the temperature-induced sol-gel transformation not only enables gentle cell encapsulation but also ensures the stability of the gel structure and cell activity (Klouda, 2015). For cultivation systems that need to adapt dynamically to the metabolic microenvironment, the pH-sensitive hydrogel can regulate the degradation rate by responding to the changes in the acidity and alkalinity of the culture environment in real-time. This characteristic makes it highly valuable in suspension cultivation systems (Schmaljohann, 2006). In cases where precise control of the solid-liquid interface is required, such as in gas-liquid interface cultivation, the photosensitive hydrogel stands out due to its controllable cross-linking properties: by irradiating with ultraviolet or blue light, the surface curing degree of the scaffold can be precisely controlled, maintaining a stable interface structure while ensuring the sufficient diffusion of nutrients in the deep layers (Fairbanks et al., 2009; Yue et al., 2015). These three types of responsive scaffolds, through different stimulus-response mechanisms, jointly construct a dynamically adjustable microenvironment system for organoid cultivation.

The introduction of biotechnological engineering has brought a revolutionary breakthrough in the cultivation of organoids. Biotechnology enables precise regulation of the microenvironment of organoids, not only enhancing their simulation accuracy but also breaking through the limitations of traditional culture methods. This allows organoids to achieve scalable expansion and cultivation while maintaining tissue specificity. Micro-pattern technology precisely controls cell adhesion and spatial arrangement through surface chemical modification or physical constraints, promoting uniform growth of organoids. For instance, Jiang et al. developed a micro-pattern agarose scaffold, which significantly outperformed the traditional matrix gel dome culture system in terms of size uniformity and cell composition for liver organoids derived from pluripotent stem cells (Jiang et al., 2022). 3D printing technology precisely regulates the mechanical properties, biochemical signals, and spatial structure of the scaffold to provide an appropriate microenvironment for organoids, promoting their growth and maturation (Ji and Guvendiren, 2017). For example, Alonzo et al. designed a ring-shaped hydrogel scaffold that not only maintains structural stability for a long time but also promotes the proliferation and maturation of cardiac organoids (Alonzo et al., 2022). Microfluidic technology precisely regulates fluid shear force, solute gradients (such as growth factors and drug concentration distribution), oxygen partial pressure levels, and mechanical stimulation (such as simulating intestinal peristalsis, vascular shear stress, etc.) to simulate the complex microenvironment in the body, thereby revealing the response mechanism of organoids to external stimuli in a dynamic microenvironment (Saorin et al., 2023). For instance, Quintard et al. developed a microfluidic platform, which not only achieved efficient encapsulation of organoids but also dynamically perfused to form functional vascular networks (Quintard et al., 2024). Bioreactor technology introduces mechanical stimulation and optimizes the culture environment, significantly enhancing the amplification efficiency and functional maturity of organoids. Compared to static culture, bioreactors can optimize nutrient and oxygen supply, reduce metabolic waste accumulation, and support the formation of vascular networks (Licata et al., 2023). For example, Ye et al. developed a micro-rotating bioreactor, which increased the proliferation rate of liver organoids by 5.2 times (Ye et al., 2024). It is worth noting that these biotechnological engineering techniques exhibit a strong synergistic effect. The combination of 3D printing and microfluidic technology can construct a heart organoid chip with vascular networks (Zhang et al., 2016); the combination of micro-pattern technology and bioreactors can achieve high-throughput organoid production (Khan et al., 2021). This multi-technology integration strategy is driving organoid models towards higher simulation accuracy and standardization, providing powerful tools for disease modeling, drug development, and regenerative medicine research.

By selecting appropriate cultivation methods, choosing organoid scaffolds that match the mechanical and biochemical properties of the target tissues, and combining them with biotechnological approaches, it is possible to more accurately simulate the tumor microenvironment and achieve the clinical translation of organoid technology. Different tumor organoids require specific cultivation methods to better simulate their *in vivo* microenvironment. For example, the air-liquid interface cultivation method provides a physiological microenvironment for tissue-air contact, offering a more similar culture system to the *in vivo* microenvironment for respiratory system models such as airway and lung organoids (Joo et al., 2024; Ekanger et al., 2025). The embedding method can provide three-dimensional structural support for organoids and simulate the mechanical properties of extracellular matrix, suitable for the cultivation of most solid tumor organoids. The mechanical and biochemical properties of the scaffolds have significant impacts on the morphology, proliferation, differentiation, and drug response of tumor organoids (Curvello et al., 2023). Studies have shown that the mechanical properties of the tumor microenvironment differ significantly from those of normal tissues. For instance, the elastic modulus of breast cancer tissue is typically 4–12 kPa, much higher than the 0.5–2 kPa of normal breast tissue (Paszek et al., 2005). Therefore, the mechanical parameters of the scaffolds need to be highly matched with the target tumor tissue to truly simulate the tumor microenvironment (Casey et al., 2017). Additionally, the porosity and three-dimensional structure characteristics of the scaffolds are related to the diffusion of nutrients and cell migration. Optimized biomimetic porous scaffolds can promote the formation of vascular networks in organoids, thereby better simulating the *in vivo* microenvironment of tumors. In terms of biochemical properties, the extracellular matrix components of the scaffolds need to simulate the molecular composition of the natural tumor microenvironment. Studies have shown that scaffolds rich in collagen are more conducive to the proliferation of breast cancer organoids, while scaffolds rich in fibronectin and laminin are more suitable for constructing the microenvironment of liver cancer organoids (Sokol et al., 2016; Saheli et al., 2018). Moreover, by precisely regulating the release of growth factors and drugs, dynamic regulation of the proliferation, differentiation, and drug response behaviors of tumor organoids can be achieved. The introduction of biotechnological approaches (such as microfluidic technology, and 3D printing technology) significantly improves the biomimetic performance of the scaffolds. Microfluidic technology precisely regulates fluid shear force, solute gradients (such as growth factor and drug concentration distribution), oxygen pressure levels, and mechanical stimuli (such as simulating intestinal peristalsis, vascular shear stress, etc.), simulating the complex tumor microenvironment *in vivo* (Sontheimer-Phelps et al., 2019). 3D printing technology precisely regulates the mechanical properties, biochemical signals, and spatial structure of the scaffolds to achieve a highly biomimetic simulation of tumor tissue heterogeneity (Wang et al., 2024). In summary, by optimizing the organoid cultivation system, not only can the reliability of cancer mechanism research and drug screening be improved, but also the application of organoid technology in clinical practice can be promoted.

## 3 Common organoid scaffold materials

### 3.1 Matrigel

**FIGURE 2:**
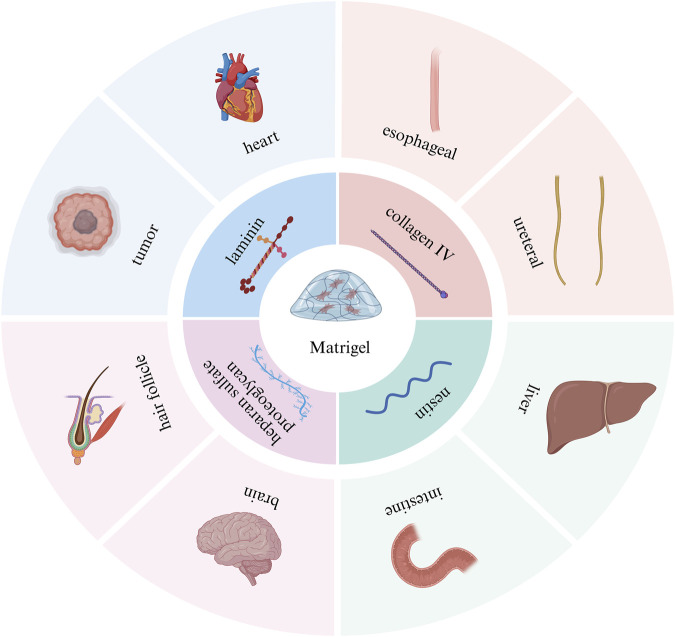
Introduction to Matrigel scaffolds. The main components and application examples of Matrigel scaffolds. This picture was created using the drawing software BioRender.

Matrigel is derived from Engelbreth-Holm-swarm mouse sarcoma and is rich in ECM proteins. Proteomics analysis showed that Matrigel contains more than 1800 kinds of proteins (Hughes et al., 2010). The main components of Matrigel include laminin, collagen IV, nestin, and heparan sulfate proteoglycan (Aisenbrey and Murphy, 2020), which are ubiquitous in early embryonic development, so Matrigel can mimic the microenvironment of early embryos. Matrigel has become the gold-standard biomaterial for establishing three-dimensional tissue models across multiple organ systems, such as tumor organoids (Qiang et al., 2023; Berk, 2022), heart organoids (Drakhlis et al., 2021), esophageal organoids (Ko et al., 2022), ureteral organoids (Shi et al., 2023), and liver organoids (Takebe et al., 2013) (Figure 2). In addition, Matrigel was used for almost all organoids successfully cultured for the first time. For example: In 2009, Sato et al. isolated crypts from the small intestine of mice and mixed them with Matrigel. Subsequently, the mixed suspension was seeded on culture plates using the dome method, and a small intestinal organoid medium containing factors such as R-spondin 1, EGF, and Noggin was added. Finally, intestinal organoids were successfully grown. Their findings validated that individual Lgr^5+^ intestinal stem cells possess the capability to generate intestinal organoids (Sato et al., 2009); In 2013, Lancaster et al. first induced human pluripotent stem cells (hPSCs) to form embryoid bodies in diluted Matrigel-coated plates. These embryoid bodies were further differentiated into neural structures in a neural induction medium, which was then encapsulated in Matrigel and transferred to a rotary bioreactor containing a differentiation medium for further induction into mature brain organoids. Brain organoids generated in this way have discrete but interconnected brain regions and exhibit features of human cortical development (Lancaster et al., 2013); In 2022, Kageyama et al. isolated epithelial and mesenchymal cells from mouse embryonic skin and cultured them in suspension in an Advanced DMEM/F-12 medium containing 1% Matrigel. Finally, hair follicle organoids were successfully induced, and the hair shaft was able to grow to 3 mm in length (Kageyama et al., 2022).

Although Matrigel has been widely used due to its many advantages, it also has many drawbacks. Given the complexity and inherent uncertainty associated with Matrigel, as well as the variability in its composition (Kleinman and Martin, 2005; Cruz-Acuna and Garcia, 2017; Polykandriotis et al., 2008), the biochemical characteristics of Matrigel can differ not only between batches but also within the same batch. For example, compositional uncertainty. One study showed that growth factors such as IGF-1 and EGF were expressed at quantifiable levels, on the order of nanograms per milliliter, but were not detected in four later independent Matrigel batches (Vukicevic et al., 1992). At the same time, the concentration of growth factors varied between batches, for example, the concentration of FGF-2 and platelet-derived growth factors varied by orders of magnitude between batches; Uncertainty of mechanical properties. A previous study showed an average elastic modulus of 400–420 Pa for two batches of Matrigel. However, the average elastic modulus of the third batch of material was twice as large (840 Pa) (Soofi et al., 2009). Finally, Matrigel is derived from mouse tumor cells, has potential immunogenicity, and its complex composition makes it difficult to fully mimic the microenvironment of specific human tissues, which limits its application in clinical transplantation and regenerative medicine.

### 3.2 dECM hydrogel scaffolds

**FIGURE 3:**
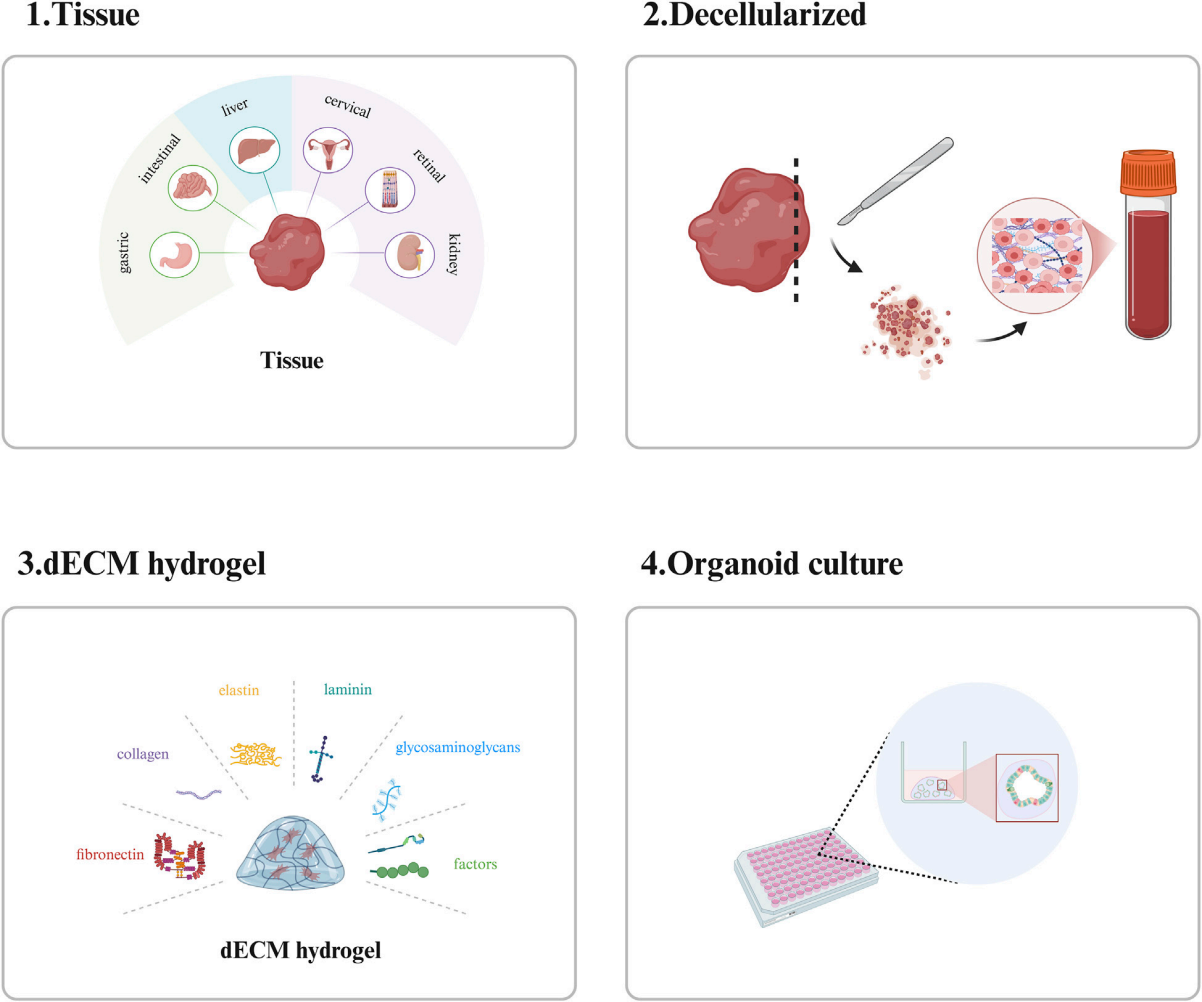
Introduction to dECM hydrogel scaffolds. The preparation, main components and applications of dECM hydrogel scaffolds. This picture was created using the drawing software BioRender.

dECM hydrogel scaffolds can be used to construct scaffold materials suitable for organoid growth by removing cellular components from tissues or organs and retaining their ECM. The ECM not only contains bioactive substances required for organoid growth but also can provide mechanical support for it. Its components contain a variety of proteins, such as fibronectin, collagen, elastin, and laminin, and also contain GAGs and a variety of bioactive factors, which play an important role in organoid culture (Navaee et al., 2023) (Figure 3). There are variations in the composition of the extracellular matrix (ECM) across different tissues, and these variations enable the ECM to meet the specific functional demands of each tissue type. Consequently, the composition of decellularized ECM (dECM) hydrogel scaffolds varies among different tissues. For instance, cardiac decellularized scaffolds primarily consist of collagen, non-collagenous proteins, elastin, proteoglycans, and glycosaminoglycans. Among these components, collagen is crucial for maintaining the mechanical strength and structural integrity of the heart, whereas elastin and proteoglycans contribute significantly to preserving the elasticity and functionality of the heart (Simsa et al., 2021). Unlike brain tissue-derived decellularized scaffolds, liver-decellularized scaffolds are mainly composed of collagen, laminin, fibronectin, and GAGs. Among these components, collagen and GAGs play a key role in maintaining the structure and function of the liver, while laminin and fibronectin promote the adhesion and proliferation of hepatocytes (Liu et al., 2025).

Currently, there are many tissue types of dECM hydrogel scaffolds for organoid culture, for example: Kim et al. prepared gastrointestinal organoid scaffolds from decellularized treated stomachs and small intestines. Compared with Matrigel, this scaffold contains more proteins derived from native gastric and intestinal tissues, which are involved in gastrointestinal-specific functions such as digestion, intestinal absorption, and gastric acid secretion, indicating that this scaffold can provide a microenvironment closer to the *in vivo* environment for gastrointestinal organoids. The results showed that the gastrointestinal organoids cultured on the scaffold were comparable to those cultured in Matrigel, in structural and functional characteristics (Kim et al., 2022a). In addition, another of his studies showed that intestinal organoid cell death was observed in acellular hydrogels derived from other tissues such as skin, lymph, heart, and muscle (Kim et al., 2022b). Together, these two studies demonstrated that the effect of the ECM microenvironment on organoid development is tissue-specific; Chen et al. fabricated a mouse-derived decellularized liver scaffold and used this scaffold to culture primary mouse cholangiocytes to successfully construct a functional liver organoid. The results show that these functional biliary organoids not only express a wealth of specific biomarkers but also exhibit a typical biliary tree-like structure with specific bile secretion and transport functions (Chen J. et al., 2023); Song et al. prepared a uterocervical extracellular matrix hydrogel from paracancerous cervical tissue from patients with cervical squamous cell carcinoma and used it for the culture of cervical squamous cell carcinoma organoids. Their results showed that compared with Matrigel, the uterocervical extracellular matrix hydrogel contained higher levels of human cervical tissue-specific proteins, the cultured organoids retained more cervical cancer-related oncogenes and signaling pathways and exhibited more obvious drug resistance, which could more accurately identify patients with drug resistance (Song et al., 2024). In addition to the above-mentioned organoids, retinal organoids (Berber et al., 2022), endodermal organoids (Giobbe et al., 2019), kidney organoids (Geng et al., 2022), etc. can also be cultured by dECM hydrogel scaffolds.

dECM hydrogel scaffolds retain the native tissue structure and components of the ECM by removing the cellular components and are therefore tissue-specific and less immunogenic. At the same time, the retained ECM components reduce the introduction of exogenous substances and avoid external interference. It has been widely used in tissue repair and medical cosmetology. However, dECM hydrogel scaffolds still have some shortcomings in organoid culture: 1. Compared with Matrigel, dECM hydrogel scaffolds still have some shortcomings in the culture effect of most organoids; 2. The quality of the stent is affected by the health status of the donor; 3. Residual chemicals and enzymes during decellularization may be cytotoxic; 4. The preparation method is complex; 5. The cells could not be completely removed; 6. There are batch differences (Talbot and Caperna, 2015). These limiting factors, to some extent, affect the widespread application of dECM hydrogel scaffolds in organoid culture.

### 3.3 Recombinant protein and peptide hydrogel scaffolds

Given the limitations of organoid scaffolds mentioned above, researchers have begun to focus on recombinant protein and peptide hydrogel scaffolds with clear components, hoping to construct fully synthetic scaffolds suitable for organoid growth. At present, the exploration of recombinant protein and peptide hydrogel scaffolds has been quite extensive.

#### 3.3.1 Recombinant protein hydrogel scaffolds

The recombinant protein hydrogel scaffold has good biocompatibility and can provide the required microenvironment for organoids by adjusting the type and concentration of protein and the mechanical and chemical properties of the scaffold (Madl et al., 2016; Dooling and Tirrell, 2016). The common recombinant proteins used in organoid scaffolds include recombinant laminin, recombinant fibrin, and recombinant elastin. In the cultivation of organoids, the recombinant protein hydrogel scaffolds are usually optimized for their performance through biotechnological methods. The main approaches include the following two. The first one involves modifying the sequence of the recombinant protein through genetic engineering, introducing specific functional domains (such as RGD adhesion peptides, matrix metalloproteinase response sites, etc.) to enhance the cell adhesion or degradation controllability of the scaffold (DiMarco et al., 2015). For instance, Kozlowski et al. prepared organoid scaffolds by inserting RGD sequences from fibrin or LAMA3 mimetic sequences into recombinant trimeric proteins. Experimental results showed that the prepared organoid scaffolds could promote the growth of endocrine lineage cells in pancreatic organoids (Kozlowski et al., 2023). The second approach involves precise control of the mechanical properties of the scaffold through cross-linking reactions and stimulus responses (such as temperature, light, and enzymes) (Liu et al., 2024). For example, Hunt et al. developed an intestinal organoid scaffold using recombinant elastin and HA. The recombinant elastin is modified with a hydrazine group and contains an RGD peptide ligand that binds to the cellular integrin receptor. However, HA was modified by the benzaldehyde functional group. The results show that the stiffness of the scaffold can be regulated by adjusting the degree of cross-linking of hydrazine and benzaldehyde. The elastoviscosity of the scaffold can be changed by replacing the benzaldehyde group on HA with an aldehyde group. Moreover, the scaffold can support the formation, growth, passage, and differentiation of intestinal organoids (Hunt et al., 2021). However, there are still many shortcomings in the application of recombinant protein hydrogels. First of all, not all proteins can be expressed in recombinant form with guaranteed refolding and function. Second, some recombinant proteins may be immunogenicity (Vigneswaran et al., 2016; Collier et al., 2010; Rosenberg, 2006; Rudra et al., 2012; Rudra et al., 2010), and that the recombinant proteins are derived from humans does not absolutely guarantee their non-immunogenicity. Finally, the production and purification of recombinant proteins are relatively complex and costly.

#### 3.3.2 Peptide hydrogel scaffolds

Peptides are composed of amino acids, are biocompatible, and do not cause cytotoxic or immune responses. By changing the peptide sequence and concentration, the mechanical properties of peptide hydrogel scaffolds can be adjusted to better mimic the mechanical properties of different tissues. Moreover, peptide hydrogel scaffolds can form a stable three-dimensional network to provide support for organoids (Dou and Feng, 2017). In organoid cultivation, peptide hydrogel scaffolds are usually optimized for their properties through biotechnological engineering. The main methods include the following three. The first one involves modulating the self-assembly peptide sequences or modifying the functional domains to enhance the mechanical and biochemical properties of the organoid scaffolds. For instance, in peptide amphiphiles (PAs) with ECM-like nanofiber structures, adding laminin motifs IKVAV and tyrosine-functionalized hyaluronic acid HA-Try to prepare brain organoid scaffolds. Studies have shown that the prepared organoid scaffolds not only exhibit biological activity but also possess nanofiber structures, and their hardness can be regulated by adjusting the concentration of HA-Tyr (Isik et al., 2023). The second one involves precise control of the mechanical properties of the scaffolds through dynamic cross-linking and stimulus-responsive mechanisms (Katyal et al., 2020). For example, Nguyen et al. designed a short peptide self-assembling hydrogel based on the tryptophan zipper (Tpzip) motif. The viscosity of this hydrogel is regulated by dynamic cross-linking, while the stiffness is regulated by temperature (e.g., the stiffness at 37°C is 10 times that at 20°C). Studies have shown that the Tpzip hydrogel scaffold can promote the growth and differentiation of human intestinal organoids (Nguyen et al., 2023). The third one involves simulating the microenvironment by loading growth factors (such as EGF/Wnt) or ECM proteins (such as laminin) (Qin et al., 2025). For example, Wan and his colleagues added type I collagen, fibronectin, and laminin to the self-assembling peptide hydrogel DRF3 to prepare colon cancer organoid scaffolds. The results showed that compared to two-dimensional cultivation, the culture time was significantly shortened, the culture efficiency was significantly improved, and the expression level of the gene MC1R related to colon cancer progression was significantly increased (Wan et al., 2022). Although peptide hydrogel scaffolds have been used in the culture of multiple types of organs, there are still some limitations. Firstly, the preparation and functionalization of peptide hydrogel scaffolds are complicated. Second, although its biological activity can be enhanced by introducing functionalized motifs, its endogenous bioactive substances are relatively limited, and it may be necessary to additionally add growth factors or other biomolecules to meet cellular requirements. In addition, long-term culture may lead to structural changes, which in turn affect cell growth and differentiation (Wulf and Bisker, 2022).

### 3.4 Synthetic hydrogel

In order to further explore the effect of the mechanical properties of scaffolds on organoid culture, researchers have turned to synthetic hydrogel scaffolds. We classified synthetic hydrogels into synthetic polymer hydrogels and natural polymer hydrogels according to the source of the synthetic materials.

#### 3.4.1 Polymeric hydrogels were synthesized

PEG, PIC, and poly (lactide-glycolide) (PLGA) are commonly used in organoid scaffolds. Among them, PEG hydrogel scaffolds have been used in the culture of various types of organs, such as intestinal epithelial organoids (Wilson et al., 2021; Zhang et al., 2020), pancreatic organoids (Shi et al., 2021), and liver organoids (Klotz et al., 2019). PEG hydrogels can adjust their mechanical properties by adjusting the concentration and molecular weight of the crosslinker, which allows them to mimic the stiffness of different tissues and thus better support organoid growth and differentiation. For example, Some researchers used a 4-arm PEG macromolecular hydrogel scaffold to cultivate neuroendocrine prostate cancer organoids and optimized the scaffold by adding bioactive substances (such as GFOGER, REDV, and RGD). The research results showed that compared with Matrigel, the 4-arm PEG macromolecular hydrogel scaffold could regulate the growth of neuroendocrine prostate cancer organoids, the expression of EZH2, and the DNA methylation status, and activate specific gene expression (Mosquera et al., 2022). Furthermore, Researchers have designed a hybrid network structure hydrogel based on the cross-linking of two different multi-arm PEG macromolecules (8-PEG-cytosine 50 and 8-arm thiol functionalized PEG): one covalently linked by Michael addition; The other is that at physiological pH, cytosine forms three hydrogen bonds with its tautomer, resulting in reversible cross-linking. The mechanical properties of gels can be modulated by dynamic rearrangement mediated by reversible hydrogen bonds. Studies have shown that this adjustable matrix can effectively relieve organoid stress and promote intestinal organoid sprouting by increasing the formation of Paneth cells (Chrisnandy et al., 2022). However, due to the ability of PEG hydrogels to inhibit protein adsorption and cell adhesion (Fairbanks et al., 2014), researchers often need to add other bioactive substances to PEG hydrogels to optimize the scaffold. For example, some researchers have added poly-D-lysine to PEG hydrogels to promote the adsorption of ECM proteins. The intestinal epithelial organoids cultured on this scaffold recapitulate the important physiological functions of the native intestinal epithelium, including multilineage differentiation and apical-basal polarization (Shi et al., 2021); They adjusted the ratio of PEG hydrogel and gelatin and the total polymer concentration to adjust the mechanical properties of the scaffold, and introduced laminin (LN111, LN521), which is related to the properties of hepatocytes, to optimize the scaffold. The results showed that the expression of related proteins and genes in the liver organoids cultured on the scaffold was comparable to that in Matrigel (Ye et al., 2020).

The network in the PIC hydrogel is more porous, which facilitates the transport of organoid nutrients and waste. Moreover, by adding other bioactive substances to the PIC hydrogel, the mechanical and biochemical properties of the scaffold can be adjusted to meet the growth needs of different types of organs. For example, some researchers have grown liver organoids using PIC hydrogel scaffolds. They optimized the scaffold by adjusting the concentration of RGD and laminin actin complex (LEC) in the scaffold and found that the concentration of RGD was 0.2 mM and LEC was 3 mg/mL, which effectively promoted the formation and proliferation of organoids. Liver organoids cultured on scaffolds were able to differentiate into hepatocyte-like phenotypes with key liver functions and maintained for more than 14 generations. In addition, LEC can be replaced with LN111 to prepare a fully synthetic hydrogel scaffold for liver organoid culture (Bealer et al., 2023). Similarly, breast organoids cultured only on RGD-modified PIC hydrogel scaffolds were shown to be able to be generated from breast fragments or single breast epithelial cells (Ye et al., 2020).

In addition to the above two polymer materials, PLGA has emerged as a prominent candidate for 3D organoid engineering, owing to its tunable erosion profiles that synchronize with tissue maturation timelines while maintaining low immunogenicity and predictable degradation kinetics—critical attributes for dynamic extracellular niche modeling. For example: Bealer et al. successfully grew pancreatic organoids in a microporous PLGA scaffold. They found that early - to mid-stage scaffold-derived β-cell progenitors of islet organoids improved glucose-stimulated insulin secretion *in vitro* compared with organoids formed at the pancreatic progenitor stage (Bealer et al., 2023); Lancaster et al. used suspension culture of brain organoids with PLGA copolymer fiber microfilaments, and the cultured brain organoids showed improved neuroectoderm formation ability and cortical development (Lancaster et al., 2017).

#### 3.4.2 Natural polymer hydrogel

A variety of natural polymers are utilized in organoid scaffolds, including sodium alginate, gelatin (Carigga Gutierrez et al., 2022), chitosan (Wang et al., 2020), and nanocellulose (Prince et al., 2022). Notably, sodium alginate and nanocellulose are the most frequently employed among these materials.

Sodium alginate has good biocompatibility and low immunity and does not cause a cellular immune response. By adjusting the concentration of sodium alginate and the type and concentration of cross-linking agents, the mechanical properties of sodium alginate scaffolds can be adjusted to meet the mechanical characteristics of different types of organs. In addition, the alginate gel can provide a good cell interface, which is conducive to cell adhesion, proliferation, and differentiation (Kang et al., 2021). These characteristics make it an ideal material for organoid scaffolds. For example: Carigga Gutierrez et al. used sodium alginate mixed with gelatin to prepare tumor organoid scaffolds. The results showed that the growth of tumor organoids on the scaffold was similar to that on the commercially available ECM scaffold (Carigga Gutierrez et al., 2022); Sen et al. used functionalized alginate beads as scaffolds to successfully grow small-cell lung cancer organoids that recapitulated the pathology and immunophenotype of the patient’s tumor (Sen et al., 2023); Sisakht et al. successfully cultivated bladder cancer organoids using alginate hydrogel scaffolds, and the gene expression profile and tissue structure characteristics of the bladder cancer organoids were highly similar to those of organoids cultured in traditional basement membrane matrix (Sisakht et al., 2025). Zhao et al. constructed a three-dimensional hydrogel scaffold suitable for liver cancer organoid cultivation using a composite system of alginate and methacrylate hyaluronic acid. They optimized the mechanical properties of the scaffold by adjusting the cross-linking density of alginate and methacrylate hyaluronic acid and improved the biochemical properties of the scaffold by introducing two adhesion molecules, methacrylamide dopamine and cyclic arginine-glycine-aspartic acid peptide. The results showed that the liver cancer organoids formed in this scaffold exhibited higher drug resistance than those cultured in traditional two-dimensional cultures (Zhao et al., 2024). Wang et al. fabricated a hydrogel scaffold with fibrin, alginate, and chitosan and used it to grow hipSC-derived liver organoids. The experimental results showed that the liver organoids cultured on this scaffold had key characteristics of the human liver and possessed liver-specific functions, such as urea synthesis and albumin secretion (Prince et al., 2022).

Nanocellulose has a high specific surface area, which can provide more adhesion sites for cells and enhance the interaction between cells and scaffolds. At the same time, its fiber network structure can not only provide support for organoids, but also network pores facilitate the exchange of nutrients and metabolic wastes (Leong et al., 2023). Due to these characteristics, nanocellulose is an ideal material for organoid scaffolds. The nanofiber hydrogel (EKGel) scaffold prepared by Prince et al., which is composed of cellulose nanocrystals and gelatin, has the advantages of two raw materials. It can not only adjust the mechanical properties but also simulate the fibrous structure of the tumor extracellular matrix, providing a good environment for the growth and proliferation of organoids. Their results showed that the breast tumor organoids grown in EKGel and BME were highly consistent in terms of tumor histopathological features, gene expression, and drug response (Kang et al., 2021). Using collagen nanocellulose hydrogel as a scaffold, Curvello et al. generated intestinal organoids that exhibited features of epithelial budding, maintained cell viability and metabolic activity, and expressed key cellular markers (Curvello et al., 2021).

The synthetic hydrogel has good batch-to-batch consistency and repeatability, which is suitable for standardized experimental operation. In addition, the biochemical and mechanical properties of synthetic hydrogels can be precisely regulated, providing a customized environment for organoid growth and differentiation to explore the effects of mechanical properties and chemical cues on cell fate (Li et al., 2014; Lee et al., 2017). However, synthetic hydrogels also have many drawbacks. First, many synthetic hydrogels require the incorporation of bioactive substances, thus increasing the cost of preparation; Second, possible degradation into cytotoxic by-products limits the types of polymers that can be used in cell culture (Kharkar et al., 2013); Third, they may contain groups that are toxic to cells; Finally, a foreign body reaction may be triggered (Anderson et al., 2008).

### 3.5 Composite scaffold

Because different types of stents have their shortcomings, researchers adopt a strategy of compositing different scaffold materials to avoid these defects. Many materials are often combined with Matrigel to prepare organoid scaffolds. For example, Matrigel was combined with a medical carbon fiber (CF) scaffold. CF scaffolds have grooves along the pores, which can effectively adsorb nutrients in the medium and promote intercellular interactions. At the same time, CF scaffolds are highly stable and do not degrade to toxic substances or cause pH changes during organoid culture. Studies have shown that the composite scaffold composed of Matrigel and CF can improve the proliferation and differentiation efficiency of iPSC in organoids (Tejchman et al., 2020); Three-dimensional recombinant spider silk microfibrous scaffolds assembled from recombinant full-length human laminin and composite with Matrigel for brain organoid culture. The results showed that the composite scaffold effectively alleviated the hypoxia problem of brain organoids and promoted the growth and differentiation of brain organoids (Sozzi et al., 2022); Composite scaffolds for culturing human spinal cord organoids (ehSC) were prepared by wrapping the ventral spinal cord signaling Shh agonist (SAG) with porous chitosan microspheres (PCSM) and coating the PCSM with a layer of Matrigel. The results showed that EHscs could form progenitor cells and neurons with significant domain specificity, construct dorsoventral spinal-like cells, and exhibit functional calcium activity (Xue et al., 2023). In addition to being composite with Matrigel, it can also be composite with other materials. For example, Gomez-Alvarez et al. mixed PM peptide hydrogel with EndoECM hydrogel to produce an organoid scaffold suitable for the growth of endometrial organoids (hEOs). The results showed that the morphology of hEOs cultured on the scaffold was similar to that in Matrigel, and the scaffold effectively promoted the formation, proliferation, and differentiation of the organoids (Gomez-Alvarez et al., 2024).

The organoid composite scaffold has many advantages, such as: 1. It has good biocompatibility and can minimize the risk of local toxicity and adverse reactions; 2. It has good mechanical properties. The mechanical properties of the scaffolds can be adjusted by combining different materials; 3. Versatility. However, organoid composite scaffolds also have some drawbacks. On the one hand, organoid composite scaffolds have potential immune responses. On the other hand, the functional composite research of composite scaffold materials needs to comprehensively consider the mutual matching between various properties, which increases the complexity and difficulty of the research (Table 1).

**TABLE 1:** The advantages and disadvantages of different scaffold materials in organoid cultivation and their applications.

Scaffold type		Main components	Advantage	Disadvantage	Application example
Matrigel		Laminin, type IV collagen, nestin, sulfated heparan sulfate proteoglycansetc.	Simulate the microenvironment of early embryos Support the cultivation of various organoids	Significant differences in components between batches or within a batch Unstable mechanical properties Potential immunogenicity Limited clinical application prospects	Intestinal organoids (Place et al., 2009), brain organoids (Lutolf and Hubbell, 2005), hair follicle organoids (Chen and Schoen, 2019) etc.
dECM hydrogel scaffolds		Tissue-specific extracellular matrix (such as collagen, laminin, elastin, GAGs, etc.)	High tissue specificity Low immunogenicity Retains the natural ECM structure and bioactive factors	The cultivation effect may not be as good as that of Matrigel The health condition of the donor affects the quality The preparation process is complex and there is residual chemical toxicity	Gastrointestinal organoids (Quintard et al., 2024), hepatobiliary organoids (Ye et al., 2024), cervical cancer organoids (Zhang et al., 2016) etc.
Recombinant protein and peptide hydrogel scaffolds	Recombinant protein hydrogel scaffolds	Recombinant laminin, recombinant fibrin, recombinant elastinetc.	Clear composition Adjustable mechanical/biochemical properties Good biocompatibility	Some proteins are difficult to be recombined and expressed It may trigger an immune response The production cost is high	Retinal organoids (Sokol et al., 2016), pancreatic organoids (Saheli et al., 2018), intestinal organoids (Sontheimer-Phelps et al., 2019) etc.
	Peptide hydrogel scaffolds	Self-assembling peptides (such as the IKVAV motif, RGD sequence, etc.)	Good biocompatibility Programmable mechanical properties Low immunogenicity	Complex preparation Limited endogenous biological activity Insufficient long-term stability	Brain organoids (Ko et al., 2022), kidney organoids (Neal et al., 2018), colon cancer organoids (Shi et al., 2023) etc.
Synthetic hydrogel	Polymeric hydrogels were synthesized	PEG, PIC, PLGAetc.	High batch consistency Mechanical properties can be precisely controlled Suitable for standardized research	Bioactive substances need to be added May degrade into toxic by-products Potential foreign body reaction	Intestinal organoids [7, 30], liver organoids [89], brain organoids [90] etc.
	Natural polymer hydrogel	Sodium alginate, gelatin, chitosan, nanocelluloseetc.	Good biocompatibility Low immunogenicity Structure can simulate natural ECM	The mechanical strength may be insufficient The degradation of some materials is uncontrollable	Tumor organoids (Soofi et al., 2009; Liu et al., 2025), liver organoids (Simsa et al., 2021), intestinal organoids (Chen J. et al., 2023) etc.
Composite scaffold		Matrigel + carbon fiber, recombinant spider silk + laminin, peptide hydrogel + EndoECMetc.	Incorporate the advantages of multiple materials Multi-functional The mechanical properties can be adjusted	Complex design Potential immune risks Difficulties in matching all components	iPSC organoids (Talbot and Caperna, 2015), brain organoids (Madl et al., 2016), spinal cord organoids (Dooling and Tirrell, 2016), endometrial organoids (DiMarco et al., 2015) etc.

## 4 Prospects

The ECM *in vivo* is a complex and ordered three-dimensional network scaffold, which plays a crucial role in regulating cell behavior and promoting tissue regeneration. Organoid scaffolds serve as a biomimetic representation of the *in vivo* ECM and are engineered to recapitulate the microenvironment essential for organoid development. The future research directions of organoid scaffolds are also discussed. Firstly, the current matrices used for the efficient cultivation of organoids have issues related to immunogenicity, which severely hinders the clinical application of organoid technology. To address this challenge, it is particularly urgent to develop new scaffold materials with naturally low immunogenicity or complete absence of immunogenicity. If the proteins used in the scaffolds are converted to human-derived proteins, it can effectively reduce immune rejection reactions and improve biocompatibility, thereby promoting the clinical application of organoids. Secondly, the ECM of different tissues and organs are significantly different in composition and microstructure (Garreta et al., 2021). Therefore, in-depth study of the interaction between cells and the matrix microenvironment, and preparation of tissue-specific organoid scaffolds have become one of the important directions for its future development. Finally, *in vivo*, ECM undergoes dynamic changes during cell growth, which include continuous production, degradation, and remodeling of components. The dynamic balance of ECM components is essential for maintaining homeostasis in the extracellular environment (LeSavage et al., 2022). However, organoid scaffolds still have shortcomings in accurately simulating the dynamic changes *in vivo*, so the preparation of dynamic organoid scaffolds will be a major challenge in the future. In the future, the design of organoid scaffolds must comprehensively consider key factors such as immunogenicity, tissue specificity of the scaffolds, and dynamic adjustability. The core goal is to develop scaffolds that can accurately simulate the microenvironment of organoids, and improve the application potential of organoids in the fields of drug sensitivity testing, regenerative medicine, and *in vivo* transplantation.
